# The home medication review (HMR) checklist: development, validation, and feasibility study

**DOI:** 10.3389/fphar.2026.1792080

**Published:** 2026-03-23

**Authors:** M. Radhakishan, V. K. Sashindran, Adithi Kellarai, Juno Jerold Joel, S. Nanjesh Kumar, L. Ananthesh, Anjusha Alex, Uday Venkat Mateti

**Affiliations:** 1 Department of Pharmacy Practice, NGSM Institute of Pharmaceutical Sciences (NGSMIPS), NITTE (Deemed to be University), Mangalore, India; 2 Department of General Medicine, Symbiosis Medical College for Women, Symbiosis International (Deemed University), Pune, India; 3 Department of General Medicine, KS Hegde Medical Academy (KSHEMA), NITTE (Deemed to be University), Mangalore, India; 4 Department of Community Medicine, KS Hegde Medical Academy (KSHEMA), NITTE (Deemed to be University), Mangalore, India; 5 Department of Community Medicine, College of Medicine, Gulf Medical University (GMU), Ajman, United Arab Emirates; 6 Thumbay Institute of Population Health, Gulf Medical University (GMU), Ajman, United Arab Emirates

**Keywords:** home medication review, HMR, checklist, development, validation, clinical pharmacy, medication safety, pharmacist-led care

## Abstract

**Introduction:**

The Home Medication Review (HMR) is a structured, patient-centred service provided by pharmacists to facilitate the optimisation of medication therapy, mitigate drug-related issues, and improve health outcomes. Despite the global adoption of HMR, there is a lack of standardisation. To overcome this gap, a detailed HMR checklist was developed to ensure that all key elements of an HMR visit are covered. The aim was to develop and validate a comprehensive checklist to standardise the delivery of HMR and facilitate its widespread implementation in the Indian healthcare system, which can also be adopted internationally with regional modifications.

**Methods:**

A detailed literature review was conducted to develop a preliminary checklist framework, which was subsequently refined through input from a seven-member advisory board. Content Validity Index (CVI) and modified kappa statistics (k*) were used to evaluate content validity by eleven experts. The checklist was tested for its usability in the home care environment through pharmacist-conducted reviews of medications in a feasibility study involving 20 participants.

**Results:**

The 12-domain, final 65-item HMR Checklist demonstrated high levels of content validity, with an average Cronbach’s alpha (Ave-CVI) of 0.97, indicating high relevance and simplicity. In the case of Universal Agreement-CVI, the values were 0.78 and 0.75. The level of pharmacist compliance during the feasibility tests showed a high level of adherence (93.5% total compliance), with key areas demonstrating adherence at every visit. Further examination of the I-CVI and k* values was very high in all domains, demonstrating good item-level validity. Overall, the feasibility data demonstrated high patient acceptance and easy integration into the workflow.

**Conclusion:**

Overall, content validity indices and content validation processes are considered crucial factors in the development of an instrument. The study demonstrates that the HMR Checklist is a reliable, holistic, and practical instrument that can be used to standardise and implement home medication reviews, led by clinical pharmacists. It has a strong potential to enhance medication safety and provides a solid foundation for future large-scale evaluations globally.

## 1 Introduction

The Home Medication Review (HMR) is a clearly patient-centred, cooperative care service provided to the community by pharmacists (Basheti et al., 2018; Patounas et al., 2021). Its main aim is to improve the quality of medicine utilisation by improving the knowledge of patients on their medication plans (Chandrasekhar et al., 2019; Srinivas et al., 2021; Carter et al., 2013). The core objective of this service is to get the best out of the medications they are prescribed and actively avoid the possible medication issues at their home (Lee et al., 2023; Seth et al., 2025). It is an important philosophical and practical development in the field of pharmacy that focuses on the delivery of a critical professional service, in partnership with general practitioners, in addition to dispensing (Van et al., 2011; Castelino et al., 2010a). The concept also has several other names globally, including Domiciliary Medication Management Review in Australia and Medication Therapy Management (MTM) in the USA (Basheti et al., 2018; Van et al., 2011; Costa et al., 2015).

Conducting HMR in a systematic way can detect, prevent, and successfully resolve any real or potential medication issues, with pharmacotherapy intentions being optimised and the results of patient health improvement in residential settings (Deidun et al., 2019; Kouladjian O’Donnell et al., 2021). By mitigating medication-related complications, HMR can ensure the safe, effective, and appropriate use of medicines, resulting in a direct positive impact on the quality of life and health of patients. This individualised practice is especially useful for those who cannot easily access a conventional medical environment on a regular basis (Weir et al., 2019; Lenaghan et al., 2007). The growing incidence of polypharmacy, particularly in people taking numerous drugs prescribed by different doctors, increases the occurrence of adverse drug reactions, drug interactions, and increased expenditures, which may greatly affect the quality of life (Chandrasekhar et al., 2019; Castelino et al., 2010a; Papastergiou et al., 2019). Thus, HMR is crucial in ensuring safe and effective medication use in complex situations by identifying and addressing these challenges (Deidun et al., 2019).

The pharmacist-led HMR has demonstrated several benefits worldwide (Rosli et al., 2021). For instance, HMR has been found to significantly lower Drug Burden Index (DBI) scores in Australia (Kouladjian O’Donnell et al., 2021). At the same time, there was an improvement in prescribing appropriateness, characterised by a significant reduction in the scores of the Medication Appropriateness Index (MAI) (Castelino et al., 2010b). In China, the HMR interventions were associated with a statistically significant decrease in the average number of drug-related problems (DRPs), from an average of 0.88 per patient to 0.4, and a significant rise in medication adherence (Akkawi, 2023; Zhang et al., 2022). Such positive effects also spread to health-related quality of life (Basheti et al., 2018; Rosli et al., 2021; Zhang et al., 2022).

However, despite the evident strengths and the positive results, the popularity and regular adoption of HMR services are relatively low in most countries, including India (Chandrasekhar et al., 2019; Van et al., 2011). Widespread lack of knowledge on HMR may be attributed to the nascent state of clinical pharmacy and community pharmacy, and an insufficient understanding of the holistic role of pharmacists within the wider healthcare system (Tunggul Adi et al., 2021). Moreover, introducing HMR faces difficulties, largely due to the lack of standardisation. A regional outlook, such as that observed in Indonesia, suggests that a substantial proportion of community pharmacists are not yet adequately equipped to deliver in-depth, patient-centric services that are crucial to the success of HMR (Tunggul Adi et al., 2021). This health system problem is further compounded by the lack of inherently embraced, structured and evaluated standards of home health services (Patounas et al., 2021).

Even though there is evidence of conducting HMR, there is a lack of standardisation in how it is carried out, which may vary slightly by region and population. This study aimed to systematically address these critical gaps and facilitate the effective, standardised, and widespread adoption of HMR services by developing and validating a Home Medication Review (HMR) checklist. The checklist can also be utilised internationally with regional modifications.

## 2 Materials and methods

### 2.1 Conception and development of the HMR checklist

A comprehensive search was conducted to identify all pertinent literature published regarding the HMR. Four major databases were searched, including PubMed, Scopus, Embase, and Web of Science (Refer to the Supplementary File 2 for the Search strategy). Duplicate entries were eliminated, and articles about HMR and all internationally accessible guidelines on HMR were reviewed sequentially to develop a framework for creating the checklist.

#### 2.1.1 Development advisory committee

A seven-member committee was formed to provide professional advice on developing a checklist. The committee consists of two physicians specialised in general medicine, one physician specialised in community medicine, one clinical pharmacist, one community pharmacist, and two academic pharmacists.

#### 2.1.2 Development of the checklist

The initial framework was developed based on a comprehensive literature review. It was then submitted to the Development Advisory Committee, where the checklist underwent iterations to achieve the desired format. All versions were meticulously developed by taking into consideration the recommendations and feedback from the committee to make them align with the current international HMR recommendations and Pharmacy practice regulations by the Pharmacy Council of India.

The HMR Checklist comprises 12 key areas and 65 specific items. Its essence is to standardise the HMR practice. When HMR visits are conducted in the Indian healthcare service, it must be considered that all necessary elements of review are taken care of. This tool may allow pharmacists to conduct HMR systematically.

Further process is divided into patient engagement and administration preparation, overall gathering of the patient’s health and medication history; the detailed evaluation of the current medication regimen used by the patient; identifying any medication-related issues; the intensive provision and counselling of the patient; and the final recording of the results, communication with the medical facility and future arrangements of patient support.

### 2.2 Psychometric properties of the HMR checklist: a content validity analysis

Content validity is conducted to ensure that a tool measures what it is intended to do with both fulfilment and accuracy in capturing every aspect of a particular construct (Almanasreh et al., 2019). It plays a pivotal role in the development of instruments as it gathers evidence on the extent to which the items of an instrument are reflective of the intended construct of a given purpose. The analysis of the content validity is often a three-step process that includes development, judgment, quantification, and revision of the analysis. This systematic process typically encompasses (Basheti et al., 2018) a development stage (e.g., domain identification, item generation), (Patounas et al., 2021), a judgment and quantification stage (e.g., expert rating using indices like Content Validity Index or Content Validity Ratio), and (Chandrasekhar et al., 2019) a subsequent revising and reconstruction stage (Almanasreh et al., 2019; Zamanzadeh et al., 2015).

#### 2.2.1 Expert panel for validation

The content validation study was conducted by a team of eleven experts, comprising seven national experts from India and four international experts. The panel composition consisted of five academicians and research members (two academicians, one clinical pharmacologist, a postdoctoral researcher (with HMR experience), and a research coordinator), three physicians specialising in general and community medicine, two practising pharmacists in clinical and community settings, and one regulatory affairs expert. The panel consisted of experts with at least 5 years of experience in their field of speciality.

#### 2.2.2 Validation

A validation study was conducted with the panel of subject matter experts to confirm the checklist’s content validity. To achieve this, an electronic content validity assessment form was created based on the guidelines of the Uniform Electronic Transactions Act (UETA), the Electronic Signatures in Global and National Commerce Act (E-Sign Act). (United States, 2021) The form initiates with an introduction that explains the justification for developing the checklist and the objectives of the validation research. Further, professionals were asked to rate each of the items on the checklists on the relevance and clarity dimensions on a 4-point Likert-type scale (1 = Not Relevant/Clear; 2 = Item needs some revision; 3 = Relevant/Clear but needs minor revision; 4 = Very Relevant/Clear) (Zamanzadeh et al., 2015). A 4-point scale is frequently used because it does not assume a neutral midpoint and gives enough data to perform a valuable analysis. Besides the quantitative ratings, the experts were encouraged to give general comments and enhancements on every item.

### 2.3 Data analysis

When expert submissions were received, data were extracted from secure, electronically locked files. Statistical analysis was then performed on the data to establish the content validity of the checklist. The evaluation of content validity index included important indices: the item-level CVI (I-CVI), the scale-level average CVI (Ave-CVI), the universal agreement CVI (UA-CVI), and the modified kappa statistic (k*) to adjust for chance agreement.

#### 2.3.1 Item-Content Validity Index (I-CVI)

The I-CVI was calculated for each item by dividing the number of experts who rated the item as a 3 or 4 (relevant/clear) by the total number of experts. This strategy is recommended because it is centred around the quality of the items and not the performance of the experts. An I-CVI of 0.78 and above is considered to show great validity in content (Almanasreh et al., 2019).

#### 2.3.2 Scale-Content Validity Index (Ave-CVI)

The Ave-CVI, which represents the content validity of the entire tool, was calculated by averaging the I-CVI values for all items. This approach is recommended as it focuses on the quality of the items rather than the performance of the experts. An Ave-CVI of 0.90 or higher is considered to indicate excellent content validity (Almanasreh et al., 2019).

#### 2.3.3 Universal Agreement CVI (UA-CVI)

The UA-CVI was calculated by determining the proportion of items that were rated as relevant (a score of 3 or 4) by all experts. Although it is a more conservative measure, its reporting with the Ave-CVI gives a more complete view of the content validity (Almanasreh et al., 2019).

#### 2.3.4 Modified kappa statistic (k^∗^)

In order to consider the probability of chance agreement, a modified kappa statistic (k*) was computed on each of the I-CVI of all the items. The kappa statistic was used to determine the percentage of agreement, more than what would be due to chance. Probability of chance agreement (pc) was calculated by using the formula: pc = [N!÷A! (N−A)!]∗0.5^N^, where N is the number of experts, and A is the number of experts who agree that the item is relevant. Then, the modified kappa was calculated as: k∗=(I−CVI−pc)/(1−pc). Usually, the interpretation of kappa values is as follows: 0.40–0.59 (fair), 0.60–0.74 (good), and >0.74 (excellent). After data analysis, the checklist was finally revised using the quantitative indicators and qualitative feedback of the expert panel (Almanasreh et al., 2019).

### 2.4 Feasibility study

An authorised clinical pharmacist conducted a feasibility study based on the use of the checklist during home visits. The purpose of the feasibility research was to pilot the practical application and the procedures of the already validated HMR Checklist in the Indian healthcare setup. Within a 1-month period, possible respondents were identified in local health centres and hospitals, meeting the inclusion criteria of having polypharmacy, and excluding those who refused to participate.

**FIGURE 1:**
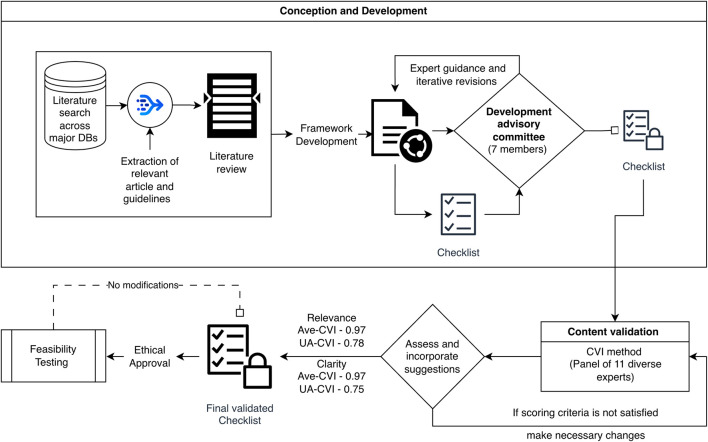
Methodology Flowchart (alt text- Image of a flowchart explaining the entire methodology process). Note: DBS, Databases; Ave-CVI, Scale-Content Validity Index (Average); UA-CVI, Universal Agreement CVI.

The methodology is explained in detail through a flowchart in Figure 1. The final validated checklist is provided as Supplementary File 1.

### 2.5 Ethics approval

This study was conducted in accordance with the principles outlined in the Declaration of Helsinki.

This study was conducted with the approval of the Nitte (Deemed to be University) Central Ethics Committee (CEC), NU/CEC/2025/935, under Study No. CEC-408, to conduct feasibility testing on participants. Also, registered with the Clinical Trials Registry - India (ICMR-NIMS), Reg. No.- CTRI/2025/08/093229 on 19-08-2025.

## 3 Results

A panel of eleven experts quantitatively evaluated the content validity of the 65-item HMR Checklist. The review was carried out on two important dimensions: relevance and clarity (Zamanzadeh et al., 2015).

The overall index of scale-level content validity (calculated as the average of all item-level CVIs (Ave-CVI)) was 0.97 in terms of both relevance and clarity, which made the tool excellent in terms of content validity. The more conservative universal agreement CVI (UA-CVI), which measures the percentage of items rated as valid by all experts, was 0.78 in relevance and 0.75 in clarity. The indices of detailed content validity of each of the 12 domains are shown in Table 1. The table provides a general overview of the item-level CVI (I-CVI) and the revised kappa statistic (k*) of all items in each domain and successfully summarises the domain-specific validity of the checklist.

**TABLE 1:** Summary of Content Validity Indices by Domain (n = 65 items).

Domain	Number of items n = 65	Relevance Ave-CVI - 0.97UA-CVI - 0.78		Clarity Ave-CVI - 0.97UA-CVI - 0.75	
		Range of I-CVI	Rage of k*	Range of I-CVI	Range of k*
1	3	0.91-0.91	0.91-0.91	0.91-0.91	0.91-0.91
2	2	0.82-0.91	0.81-0.91	0.82-1.00	0.81-1.00
3	5	0.82-1.00	0.81-1.00	0.82-1.00	0.81-1.00
4	6	0.82-1.00	0.81-1.00	0.82-1.00	0.81-1.00
5	4	1.00-1.00	1.00-1.00	1.00-1.00	1.00-1.00
6	6	1.00-1.00	1.00-1.00	1.00-1.00	1.00-1.00
7	9	0.91-1.00	0.91-1.00	0.82-1.00	0.81-1.00
8	6	1.00-1.00	1.00-1.00	0.91-1.00	0.91-1.00
9	14	0.73-1.00	0.70-1.00	0.73-1.00	0.70-1.00
10	3	1.00-1.00	1.00-1.00	0.91-1.00	1.00-1.00
11	2	1.00-1.00	1.00-1.00	1.00-1.00	1.00-1.00
12	5	1.00-1.00	1.00-1.00	1.00-1.00	1.00-1.00

I-CVI, Item-Content Validity Index; k* = Modified Kappa Statistic; Ave-CVI, Scale-Content Validity Index (Average); UA-CVI, Universal Agreement CVI.

Table 2 provides a complete breakdown of the I-CVI and the modified kappa statistic of the individual items. These findings show a high level of content validity at the item level, and most of the items had high scores on relevance and clarity.

**TABLE 2:** Item-level content validity indices (I-CVI) and modified kappa (k*) for the HMR checklist.

Relevance						Clarity					
Item No.	I-CVI	k*	Item No.	I-CVI	k*	Item No.	I-CVI	k*	Item No.	I-CVI	k*
1	0.91	0.91	34	1.00	1.00	1	0.91	0.91	34	1.00	1.00
2	0.91	0.91	35	1.00	1.00	2	0.91	0.91	35	1.00	1.00
3	0.91	0.91	36	1.00	1.00	3	0.91	0.91	36	0.91	0.91
4	1.00	1.00	37	1.00	1.00	4	1.00	1.00	37	1.00	1.00
5	0.82	0.81	38	1.00	1.00	5	0.82	0.81	38	1.00	1.00
6	0.91	0.91	39	1.00	1.00	6	1.00	1.00	39	0.91	0.91
7	0.91	0.91	40	1.00	1.00	7	0.82	0.81	40	1.00	1.00
8	0.91	0.91	41	1.00	1.00	8	1.00	1.00	41	1.00	1.00
9	1.00	1.00	42	1.00	1.00	9	1.00	1.00	42	0.91	0.91
10	0.82	0.81	43	1.00	1.00	10	0.82	0.81	43	1.00	1.00
11	1.00	1.00	44	0.91	0.91	11	1.00	1.00	44	0.82	0.81
12	1.00	1.00	45	1.00	1.00	12	1.00	1.00	45	1.00	1.00
13	0.82	0.81	46	1.00	1.00	13	0.82	0.81	46	1.00	1.00
14	1.00	1.00	47	1.00	1.00	14	1.00	1.00	47	1.00	1.00
15	0.91	0.91	48	1.00	1.00	15	0.91	0.91	48	1.00	1.00
16	0.91	0.91	49	1.00	1.00	16	0.91	0.91	49	1.00	1.00
17	1.00	1.00	50	1.00	1.00	17	1.00	1.00	50	1.00	1.00
18	1.00	1.00	51	1.00	1.00	18	1.00	1.00	51	1.00	1.00
19	1.00	1.00	52	1.00	1.00	19	1.00	1.00	52	1.00	1.00
20	1.00	1.00	53	0.73	0.70	20	1.00	1.00	53	0.73	0.70
21	1.00	1.00	54	1.00	1.00	21	1.00	1.00	54	1.00	1.00
22	1.00	1.00	55	1.00	1.00	22	1.00	1.00	55	1.00	1.00
23	1.00	1.00	56	1.00	1.00	23	1.00	1.00	56	1.00	1.00
24	1.00	1.00	57	1.00	1.00	24	1.00	1.00	57	1.00	1.00
25	1.00	1.00	58	1.00	1.00	25	1.00	1.00	58	0.91	0.91
26	1.00	1.00	59	1.00	1.00	26	1.00	1.00	59	1.00	1.00
27	1.00	1.00	60	1.00	1.00	27	1.00	1.00	60	1.00	1.00
28	1.00	1.00	61	1.00	1.00	28	1.00	1.00	61	1.00	1.00
29	0.91	0.91	62	1.00	1.00	29	0.82	0.81	62	1.00	1.00
30	1.00	1.00	63	1.00	1.00	30	1.00	1.00	63	1.00	1.00
31	1.00	1.00	64	1.00	1.00	31	1.00	1.00	64	1.00	1.00
32	1.00	1.00	65	1.00	1.00	32	1.00	1.00	65	1.00	1.00
33	1.00	1.00	​	​	​	33	1.00	1.00	​	​	​

I-CVI, Item-Content Validity Index; k* = Modified Kappa Statistic; Ave-CVI, Scale-Content Validity Index (Average); UA-CVI, Universal Agreement CVI.

The findings show high levels of validity in all 12 domains. Domains 5, 6, 8, 10, 11, and 12 scored perfect or near-perfect levels in relevance and clarity, which demonstrates the professional agreement regarding these fundamental elements of HMR. Although Domain 9 was the biggest domain with the greatest number of items 14 in the questionnaire, it presented the broadest range of scores and its general validity was strong. The I-CVI and the k* values of each item were considered acceptable, and the last instrument was updated by the quantitative and expert qualitative index and guaranteeing that each item answers its purpose.

Out of the 27 people who were screened, the feasibility study was conducted on 20 participants, with the remaining 5 members not meeting the inclusion criteria, and 2 not consenting to participate.

Maintaining a high rate of compliance with the 65-item HMR Checklist by the pharmacist was noted. The total response rate of all 65 items on the 12 domains was 93.5%. Certain crucial elements, including the validation of participant identity (Domain 3), examination of health records (Domain 4), and recording of DRPs (Domain 8), were completed in 100% of the visits. Participant education (Domain 9) specifically involved the use of the teach-back method during education, which was recorded in 17 out of 20 visits (85% follow-through). At the end of the session, all the checklists were signed and dated by the performing pharmacist.

The average time to complete an HMR home visit, with all checklists and documentation, was 75 min (median: 70 min; interquartile range: 60–95 min). The maximum duration of the visit was 95 min, and the minimum was 60 min. The time spent on certain domains, including Participant Education (Domain 9) and Collection of Health Records (Domain 4), was 20 ± 10 min and 10 ± 5 min, respectively.

## 4 Discussion

The main finding is that the checklist has very good content validity, with an Ave-CVI of 0.97, indicating both relevance and clarity. The high level of validation is one of the strengths of this instrument. This exceeds the standard of 0.90, indicating excellent validation of the content. (Almanasreh et al., 2019). A similar value of Ave-CVI (0.97–1.00) was also observed in a study conducted by Nasiri et al. in the development and validation of a checklist for use in patient care (Nasiri et al., 2023). Such strong consensus among a wide panel of professionals proves that the items in the checklist are relevant and well-constructed for their intended use. The creation of this tool is a direct response to the identified need for a structured and standardised approach to HMR in India, where services are scarce due to a lack of patient awareness, variable procedures, and issues in professional collaboration.

Moreover, the computations of the modified kappa statistic (k*) of the individual items indicate that the high degree of inter-expert agreement did not occur by chance. Several studies on content validation, conducted by Zamanzadeh et al. and Deepak et al., have also used the modified kappa statistic (k*) to assess the degree of inter-expert agreement (Zamanzadeh et al., 2015; Deepak et al., 2025). This agreement was reached in a panel of national and international specialists in different fields, including medicine, clinical and community pharmacy, and academia, which enhances the credibility and applicability of the checklist. This strong validation indicates that the checklist can serve as a framework to inform pharmacists, reminding them of all essential components of an HMR visit, including patient interactions, data collection, documentation, and follow-up.

The results of this feasibility study suggest that the HMR Checklist can be effectively implemented and utilised in a real-life scenario to conduct HMRs by trained pharmacists within the Indian medical care system. This confirms the practicality of the developed checklist. A similar feasibility study was conducted by Venkatraman et al. to assess the practicality of implementing a novel solution for patients undergoing a specific procedure (Venkatraman et al., 2022). Recruitment rates, adherence by pharmacists, and patient acceptance were generally high. The approximate average visit time provides some essential measures for conducting a larger study. The results suggest that further substantial research is needed to determine the clinical effectiveness of HMR, with specific reference to this checklist.

This study has successfully addressed the critical gap in standardisation of HMR services within the Indian healthcare context. Creating and validating the HMR Checklist ensures that all critical elements of a complete review are fully covered. Implementing or using a checklist serves several potential benefits, including serving as a memory aid, ensuring that no steps are skipped, and mitigating the effects of general fatigue, stress, and distraction, thereby ensuring that recommended best practices are in place (Cha et al., 2019). This checklist helps the pharmacist navigate each step of an HMR visit in a systematic manner, starting with initial patient interaction and the acquisition of necessary information, and culminating in the identification of medication issues, patient education, and documentation. The creation of this checklist involved extensive consultation with the development advisory committee and expert validators. It was specifically designed to suit the needs and peculiarities of the Indian healthcare setting, while still aligning with current international HMR principles and also the Program rules for HMR provided by the Australian government.

Home medication review is a structured, pharmacist-led initiative that identifies and resolves medication-related issues in their home environment. The developed home medication review checklist is a reliable, holistic, and practical tool for routine use by healthcare professionals. The well-developed, structured design helps standardise the process of HMR by clinical pharmacists. Moreover, it provides a strong and evidence-based foundation for future large-scale implementation, evaluation, and refinement at the national level.

## Data Availability

The raw data supporting the conclusions of this article will be made available by the authors, without undue reservation.
