# Supplementary material for: The home medication review (HMR) checklist: development, validation, and feasibility study
Source: Front Pharmacol. 2026 Mar 23;17:1792080. doi: 10.3389/fphar.2026.1792080 (PMC13050876; doi:10.3389/fphar.2026.1792080)
Supplement: Supplementary file 1 [file Supplementaryfile1.pdf]

Supplementary File 1

Home Medication Review (HMR) Checklist

To be used by a Clinical Pharmacist during an HMR visit. Ensure all items are addressed.

1. PARTICIPANT IDENTIFICATION *— Skip if directly referred by General Physician*

‣ *At Health Centre(s) or Hospital(s)*

- ☐ Participant screened at health centre or hospital.
- ☐ Participant provided and explained with the Participant Information Sheet (native language).
- ☐ Informed Consent Form (ICF) provided and signed.

2. RAPPORT BUILDING & SCHEDULING *— Skip if directly referred by General Physician*

‣ *At Health Centre(s) or Hospital(s)*

- ☐ Rapport initiated and home visit scheduled with participant and/or caregiver.

3. INTRODUCTION & ENCAPSULATED RAPPORT

‣ *At Home or Residence*

- ☐ Participant identity verified against previously obtained ICF (if recruited at health centre(s) or hospital(s)).
- ☐ Clinical Pharmacist self-introduction and explained the purpose of the HMR session.
- ☐ Participant was informed about the session structure.
- ☐ Family members/caregivers identities noted.

4. COLLECTION OF HEALTH RECORDS

‣ *At Home or Residence*

- ☐ Medical and medicine history documents reviewed.
- ☐ Relevant laboratory reports reviewed.
- ☐ Prescriptions/medication orders reviewed.
- ☐ Pharmacy bills/invoices reviewed.
- ☐ All relevant information transcribed into the data collection form.

5. ASSESSMENT OF MEDICATION KNOWLEDGE, ADHERENCE & QUALITY OF LIFE

‣ *At Home or Residence*

- ☐ Assessment of Medication Knowledge data recorded.
- ☐ Assessment of Medication Adherence data recorded.
- ☐ Assessment of Quality of Life data recorded.

6. MEDICATION VISUALISATION & PILL COUNT

‣ *At Home or Residence*

- ☐ Participant asked to show current medications.
- ☐ OTC, supplements, and medications reviewed.

- ☐ Previously assessed medication adherence re-verified by the pill count method.
- ☐ Observations and discrepancies noted.
- ☐ Details about the alternate system of medicines are recorded.

7. MEDICATION RECONCILIATION

‣ *At Home or Residence*

- ☐ Previously visualised medications compared with recent prescription order(s).
- ☐ Discrepancies identified and classified: omission/addition/dose/frequency/timing.
- ☐ Details recorded in the data collection form.

8. IDENTIFICATION OF DRUG-RELATED PROBLEMS (DRPS)

‣ *At Home or Residence*

- ☐ Screened for drug–drug interactions.
- ☐ Checked for wrong drug, dose, frequency, indication, or administration errors.
- ☐ Checked for Potentially Inappropriate Medications (PIMs).
- ☐ Screened for Adverse Drug Reactions (ADRs).
- ☐ Drug duplications noted.
- ☐ DRPs documented with supporting notes.

9. PARTICIPANT EDUCATION & MEDICATION-RELATED COUNSELLING

‣ *At Home or Residence*

- ☐ Name and description of the drugs.
- ☐ The dosage form, dose, route of administration, and duration of drug therapy.
- ☐ Intended use of the drug and expected action.
- ☐ Special directions and precautions for the drug.
- ☐ Common severe side effects or adverse effects, or interactions and therapeutic contraindications that may be encountered, including their avoidance, and the action required if they occur.
- ☐ Techniques for self-monitoring drug therapy.
- ☐ Proper storage of the drugs.
- ☐ Prescription refill information.
- ☐ Action to be taken in the event of a missed dose.
- ☐ Used the teach-back method to confirm understanding.
- ☐ Answered participants' medication-related queries.

10. DOCUMENTATION & COMMUNICATION

‣ *At Home or Residence*

- ☐ HMR report prepared with summary and recommendations.
- ☐ Report communicated to treating physician.

11. FOLLOW-UP VISIT(S) SCHEDULING

▸ *At Home or Residence*

- ☐ Discussion: Schedule the upcoming HMR follow-up and assessment visits.

12. CLOSING THE SESSION & RECORDKEEPING

▸ *At Home or Residence*

- ☐ Checklist signed and dated by pharmacist.
- ☐ All documents (ICF, forms, report) are stored or archived.

PHARMACIST CREDENTIALS

**Pharmacist Name**	
**Pharmacist Registration No.**	
**Signature**	
**Date**	
