# Supplementary material for: The home medication review (HMR) checklist: development, validation, and feasibility study
Source: Front Pharmacol. 2026 Mar 23;17:1792080. doi: 10.3389/fphar.2026.1792080 (PMC13050876; doi:10.3389/fphar.2026.1792080)
Supplement: Supplementary file 2 [file Supplementaryfile2.pdf]

Supplementary File 2

Literature Review Search Strategy

The following search strings were applied across four major bibliographic databases: PubMed, Scopus, Web of Science, and Embase. Searches were conducted to identify all relevant literature on pharmacist-led home medication review services.

Database 1: PubMed

#	Search String	Results
#1	"Home Visit*" OR "House Visit*" OR "Home medication review" OR "Home medicines review" OR "HMR" OR "Home care service" OR "Home service*" OR "House Service*" OR "House call"	15,392
#2	"Pharmacists" (MeSH) OR "Community Pharmacy Services" (MeSH) OR "Pharmacist" OR "Clinical Pharmacists" OR "Clinical Pharmacist" OR "Pharmacist, Clinical" OR "Pharmacists, Clinical" OR "Community Pharmacists" OR "Community Pharmacist" OR "Pharmacist, Community" OR "Pharmacists, Community" OR "Retail Pharmacists" OR "Retail Pharmacist" OR "Pharmacist"	30,884
#3	#1 AND #2	252

Database 2: Scopus

#	Search String	Results
#1	TITLE-ABS-KEY ("Home Visit*" OR "House Visit*" OR "Home medication review" OR "Home medicines review" OR "HMR" OR "Home care service" OR "Home service*" OR "House Service*" OR "House call")	60,949
#2	TITLE-ABS-KEY ("Pharmacist" OR "Clinical Pharmacists" OR "Clinical Pharmacist" OR "Pharmacist, Clinical" OR "Pharmacists, Clinical" OR "Community Pharmacists" OR "Community Pharmacist" OR "Pharmacist, Community" OR "Pharmacists, Community" OR "Retail Pharmacists" OR "Retail Pharmacist" OR "Pharmacist, Retail" OR "Pharmacists, Retail" OR "Community Pharmacy Services" OR "Pharmaceutical Service, Community" OR "Pharmaceutical Services, Community" OR "Service, Community Pharmaceutical" OR "Services, Community Pharmaceutical" OR "Pharmacy Services, Community" OR "Community Pharmacy Service" OR "Pharmacy Service, Community" OR "Service, Community Pharmacy" OR "Services, Community Pharmacy" OR "Community Pharmaceutic Services" OR "Community Pharmaceutic Service" OR "Pharmaceutic Service, Community" OR "Pharmaceutic Services, Community" OR "Service, Community Pharmaceutic" OR "Community Pharmaceutical Services" OR "Community Pharmaceutical Service" OR "Services, Community Pharmaceutic")	116,287
#3	#1 AND #2	926

Database 3: Web of Science

#	Search String	Results
#1	"Home Visit*" OR "House Visit*" OR "Home medication review" OR "Home medicines review" OR "HMR" OR "Home care service" OR "Home service*" OR "House Service*" OR "House call"	12,902
#2	"Pharmacist" OR "Clinical Pharmacists" OR "Clinical Pharmacist" OR "Pharmacist, Clinical" OR "Pharmacists, Clinical" OR "Community	28,064

	Pharmacists" OR "Community Pharmacist" OR "Pharmacist, Community" OR "Pharmacists, Community" OR "Retail Pharmacists" OR "Retail Pharmacist" OR "Pharmacist, Retail" OR "Pharmacists, Retail" OR "Community Pharmacy Services" OR "Pharmaceutical Service, Community" OR "Pharmaceutical Services, Community" OR "Service, Community Pharmaceutical" OR "Services, Community Pharmaceutical" OR "Pharmacy Services, Community" OR "Community Pharmacy Service" OR "Pharmacy Service, Community" OR "Service, Community Pharmacy" OR "Services, Community Pharmacy" OR "Community Pharmaceutic Services" OR "Community Pharmaceutic Service" OR "Pharmaceutic Service, Community" OR "Pharmaceutic Services, Community" OR "Service, Community Pharmaceutic" OR "Community Pharmaceutical Services" OR "Community Pharmaceutical Service" OR "Services, Community Pharmaceutic"	
**#3**	#1 AND #2	221

Database 4: Embase

#	Search String	Results
**#1**	'home visit*':ab,ti OR 'house visit*':ab,ti OR 'home medication review':ab,ti OR 'home medicines review':ab,ti OR 'hmr':ab,ti OR 'home care service':ab,ti OR 'home service*':ab,ti OR 'house service*':ab,ti OR 'home visit':ab,ti	20,242
**#2**	'pharmacist':ab,ti OR 'clinical pharmacists':ab,ti OR 'clinical pharmacist':ab,ti OR 'pharmacist, clinical':ab,ti OR 'pharmacists, clinical':ab,ti OR 'community pharmacists':ab,ti OR 'community pharmacist':ab,ti OR 'pharmacist, community':ab,ti OR 'pharmacists, community':ab,ti OR 'retail pharmacists':ab,ti OR 'retail pharmacist':ab,ti OR 'pharmacist, retail':ab,ti OR 'pharmacists, retail':ab,ti OR 'community pharmacy services':ab,ti OR 'pharmaceutical service, community':ab,ti OR 'pharmaceutical services, community':ab,ti OR 'service, community pharmaceutical':ab,ti OR 'services, community pharmaceutical':ab,ti OR 'pharmacy services, community':ab,ti OR 'community pharmacy service':ab,ti OR 'pharmacy service, community':ab,ti OR 'service, community pharmacy':ab,ti OR 'services, community pharmacy':ab,ti OR 'community pharmaceutic services':ab,ti OR 'community pharmaceutic service':ab,ti OR 'pharmaceutic service, community':ab,ti OR 'pharmaceutic services, community':ab,ti OR 'service, community pharmaceutic':ab,ti OR 'community pharmaceutical services':ab,ti OR 'community pharmaceutical service':ab,ti OR 'services, community pharmaceutic':ab,ti	57,190
**#3**	#1 AND #2	478

Database	Combined Search Results (#3)
PubMed	252
Scopus	926
Web of Science	221
Embase	478
**Total (before deduplication)**	**1,877**
